# Ethylene (co) oligomerization using iminopyridyl Ni(II) and Pd(II) complexes bearing benzocycloalkyl moieties to access hyperbranched ethylene oligomers and ethylene-MA co-oligomers

**DOI:** 10.3389/fchem.2022.961426

**Published:** 2022-08-04

**Authors:** Beihang Ding, Guanru Chang, Zhengpeng Yan, Shengyu Dai

**Affiliations:** ^1^ Institute of Physical Science and Information Technology, Key Laboratory of Structure and Functional Regulation of Hybrid Materials of Ministry of Education, Anhui University, Hefei, China; ^2^ School of Chemical and Environmental Engineering, Anhui Polytechnic University, Wuhu, China; ^3^ School of Chemistry and Chemical Engineering, Key Laboratory of Inorganic Functional Material, Huangshan University, Huangshan, China

**Keywords:** Ni(II) and Pd(II) complexes, ethylene oligomerization, hyperbranched, benzocycloalkyl, ethylene-MA co-oligomers

## Abstract

Hyperbranched ethylene oligomers and polar functionalized co-oligomers synthesized *via* ethylene chain walking (co) oligomerization is a very attractive strategy. In this study, a series of dibenzhydryl iminopyridyl ligands with benzocycloalkyl and naphthyl moieties and the corresponding Ni(II) and Pd(II) complexes were synthesized and characterized. The Ni(II) complexes were highly effective in ethylene oligomerization and ethylene oligomers with hyperbranched microstructures were generated from this system. The corresponding Pd(II) complexes showed moderate oligomerization activities in ethylene oligomerization and hyperbranched ethylene oligomers were also yielded from the system. More significantly, the Pd(II) complexes can also effectively promote the co-oligomerization of ethylene with methyl acrylate (MA) to obtain hyperbranched polar functionalized ethylene-MA co-oligomers. The reaction temperature, catalyst ligand structure and metal type all have significant effects on ethylene (co) oligomerization with respect to catalytic activity, molecular weight and topology of the oligomers.

## Introduction

**CHART 1:**
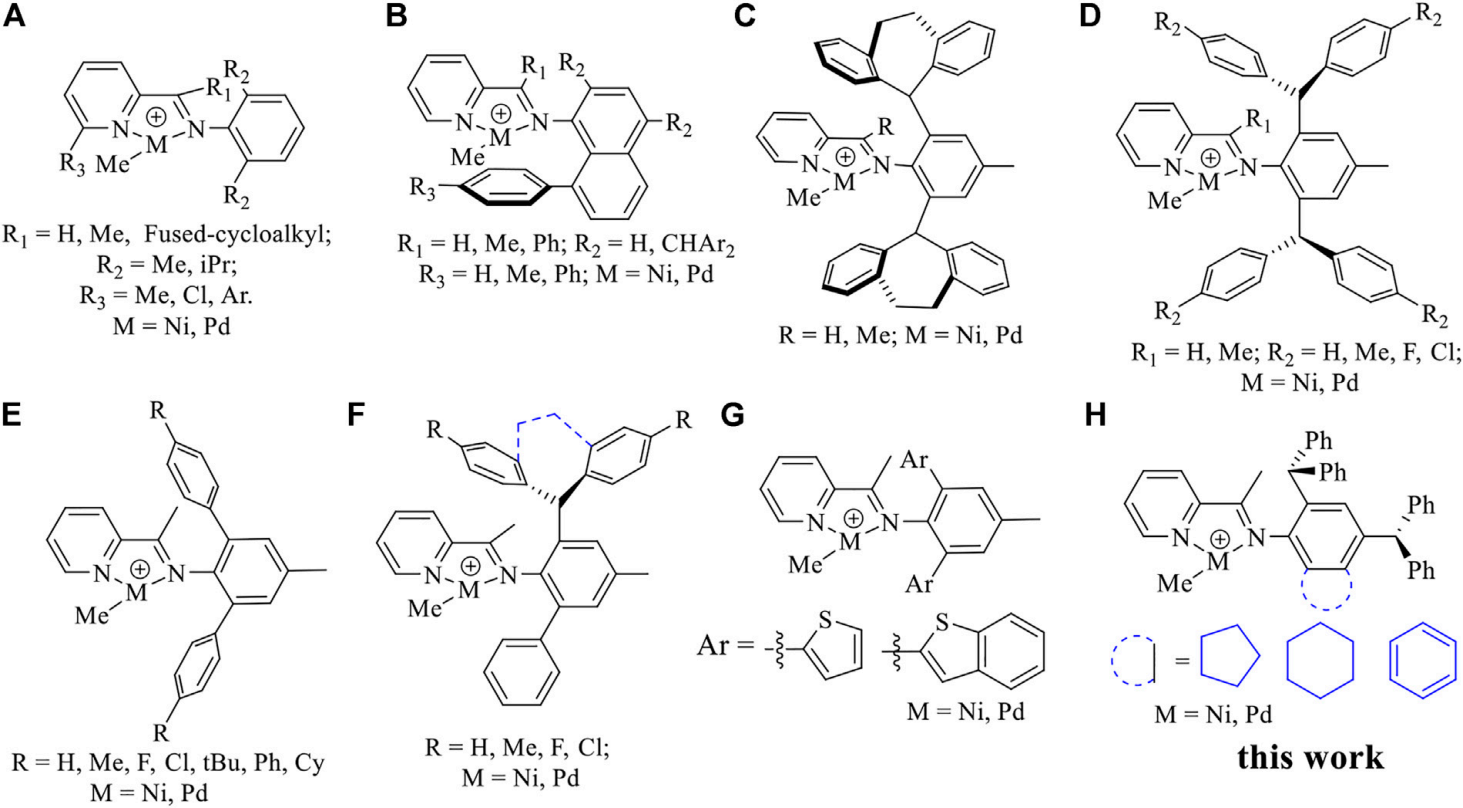
Representative iminopyridine Ni(II) and Pd(II) catalysts **(A–G)**, and new complexes described in the current work **(H)**.

The ethylene chain walking (co) oligomerization is a very attractive strategy for the direct synthesis of hyperbranched ethylene oligomers and polar functionalized co-oligomers (Stephenson et al., 2014; Wiedemann et al., 2014; Falivene et al., 2018; Mecking and Schnitte, 2020). Over the years, an impressive research effort has been conducted to rationally design ligands that enable a better control of the ethylene oligomerization and on the resulting oligomer microstructure (Tomov et al., 2006; Junges et al., 2007; Mukherjee et al., 2009; Albahily et al., 2011; Alzamly et al., 2013; Yang et al., 2014; Hameury et al., 2015; Luo et al., 2016; Liu et al., 2017; Nifant’ev et al., 2018; Sydora, 2019; Olivier-Bourbigou et al., 2020; Yeh et al., 2021). In particular, a few late-transition metal catalysts have made important advances in the preparation of branched ethylene oligomers *via* the chain walking mechanism. For example, a few N,N-iminopyrrolyl and N,O-salicylaldiminato neutral Ni(II) catalysts with specially designed ligands (Stephenson et al., 2014; Wiedemann et al., 2014; Falivene et al., 2018; Mecking and Schnitte, 2020; Cruz et al., 2021) and cationic Ni(II)- and Pd(II)-α-diimine catalysts with minor steric hindrance (Xiang et al., 2011; Meduri et al., 2013; Guo et al., 2019a) were used in the synthesis of hyperbranched ethylene oligomers and hyperbranched ethylene-based polar functionalized co-oligomers. Lately, a series of novel iminopyridyl Ni(II) and Pd(II) catalysts have also been developed to yield hyperbranched ethylene oligomers and ethylene-based co-oligomers (D'Auria et al., 2017; Saki et al., 2020; Li et al., 2021; Yan et al., 2021). Compared to the widely used α-diimine systems, which are typically used to prepare polyethylene or ethylene-based copolymers of high molecular weights, these iminopyridyl catalysts possessing a unilateral steric hindrance are known for the synthesis of low molecular weight polyethylene (Chart 1A). (Laine et al., 1999; Meneghetti et al., 1999; Laine et al., 2000; Bianchini et al., 2010) Many improvements in terms of catalytic activity, thermal stability and properties of the resulting polymer have been made *via* the introduction of bulky ortho-aryl substituents, modification of the pyridine backbone and electronic tuning (Chart 1) (Yue et al., 2014; Huang et al., 2015; Chen et al., 2016; Dai et al., 2016; Huang et al., 2016; Chen et al., 2018; Guo et al., 2019b; Dai and Li, 2020; Ge et al., 2021; Li and Dai, 2021; Peng et al., 2021; Fan et al., 2022a; Fan et al., 2022b; Yan et al., 2022a). Among all the modified catalysts, a series of half “sandwich” iminopyridyl Ni(II) and Pd(II) complexes bearing an 8-aryl-naphthyl substituent (Chart 1B) and rotation-restricted iminopyridyl Ni(II) and Pd(II) complexes with dibenzosuberyl groups (Chart 1C) can effectively suppress chain transfer in ethylene polymerization to yield high-molecular-weight polyethylene and ethylene-based copolymers (Chen et al., 2016; Dai et al., 2016; Dai and Li, 2020; Ge et al., 2021; Li and Dai, 2021; Peng et al., 2021). In contrast, N-teraryl iminopyridyl Ni(II) and Pd(II) catalysts with weak neighboring group interactions were prone to simultaneous chain walking and chain transfer reactions, thus facilitating the synthesis of hyperbranched ethylene oligomers and ethylene-MA co-oligomers (Charts 1E,G) (Fan et al., 2022a; Fan et al., 2022b; Yan et al., 2022a). More interestingly, the hybridized form of the mentioned above two types (Charts 1C,E) was also highly effective in suppressing chain transfer in ethylene polymerization to yield high-molecular-weight polyethylene (Chart 1F) (Ge et al., 2021).

In the present study, we designed and synthesized a series of iminopyridyl Ni(II) and Pd(II) complexes (Chart 1H) with benzocycloalkyl and dibenzhydryl moieties. These new iminopyridyl complexes exhibited superior performance in ethylene (co)oligomerization.

## Results and discussion

### Synthesis and characterization of iminopyridine nickel and palladium complexes

**SCHEME 1:**
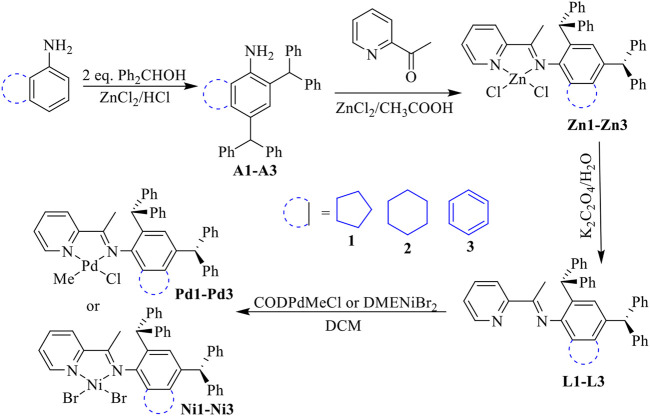
Synthesis of unsymmetrical dibenzhydryl iminopyridine ligands with benzocycloalkyl or naphthyl moieties and the corresponding iminopyridyl Ni(II) and Pd(II) complexes.

**FIGURE 1:**
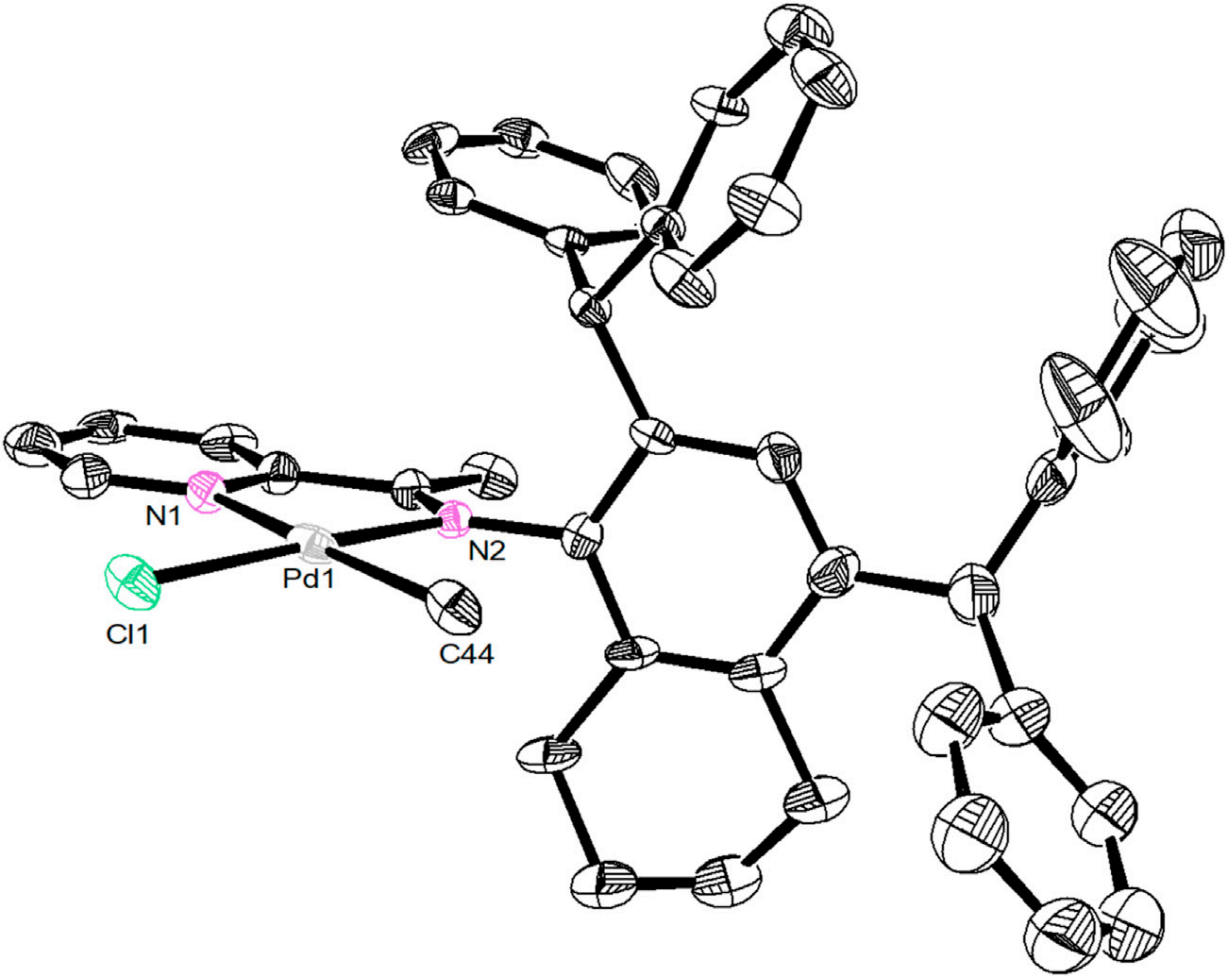
A view of the solid-state molecular structure of **Pd2** (2164023). Ellipsoids are drawn at the 30% probability level and hydrogen atoms are omitted for clarity.

Unsymmetrical bulky dibenzhydryl anilines **A1**-**A3** containing benzocycloalkyl or naphthyl moieties were synthesized *via* condensation with 2 eq. of benzhydrol in the presence of zinc chloride and hydrochloric acid (Scheme 1). The anilines were obtained in high yields (83–90%) with no need for chromatographic purification and were characterized by using ^1^H and ^13^C NMR (Supplementary Figures S1–S4) and mass spectrometries (Supplementary Figures S15,S16). Further condensation with 2-acetylpyridine using the template-type method afforded the iminopyridine ligands **L1**-**L3** (Scheme 1). (Rosa et al., 2008; Guo et al., 2018) The ligands were also prepared in high yields (69–83%) with no chromatography involved and were characterized by using ^1^H and ^13^C NMR (Supplementary Figures S5–S8) and mass spectrometries (Supplementary Figures S17,S18). Ni(II) complexes **Ni1**-**Ni3** were obtained in excellent yields (88–96%) by reaction with one equivalent of [NiBr_2_(DME)] (DME = dimethoxyethane) (Scheme 1). The purity and identity of complexes **Ni1**-**Ni3** were examined by elemental analyses and MALDI-TOF MS. Similarly, the corresponding Pd(II) complexes **Pd1**-**Pd3** were synthesized in outstanding yields (90–92%) by exposing the ligands **L1**-**L3** to PdClMe(COD) (COD = 1,5-cyclooctadiene) (Scheme 1). The purity of the obtained Pd(II) complexes was verified by ^1^H and ^13^C NMR spectrometry (Supplementary Figures S9–S14), elemental analyses and MALDI-TOF MS. The coordination of the palladium precursor shifts the positions of the characteristic peaks in the ^1^H and ^13^C NMR spectra of the corresponding ligands. Due to the electron-absorbing nature of the palladium metal, most of these characteristic peaks are shifted to lower fields. More interestingly, due to the asymmetry of the palladium precursors, the resulting complexes have two isomers with different ratios (cf. ESI). Single crystals of complex **Pd2** were obtained by layering its CH_2_Cl_2_ solution with hexanes at room temperature. As shown in Figure 1, the **Pd2** complex adopts an approximate square-planar geometry around the palladium center and the phenyl groups in the dibenzhydryl substituent deviate from the axial position of the palladium center. This is responsible for the facile chain transfer reaction observed during the polymerization. In addition, the benzocyclohexyl group exhibits a sterically distorted conformation to provide a more favorable steric environment.

### Ni(II) complexes catalyzed ethylene oligomerization

**FIGURE 2:**
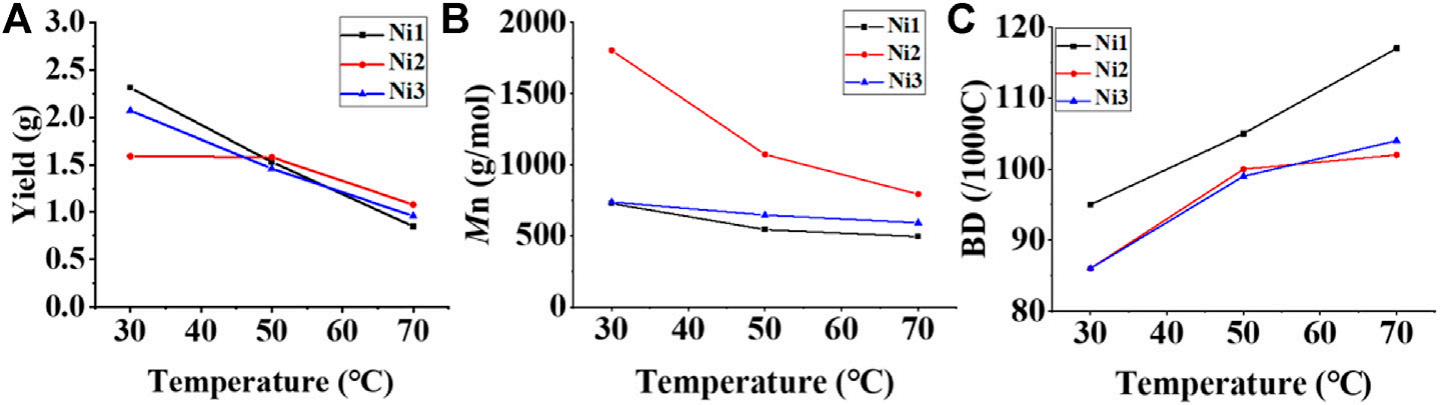
Plots of yield **(A)**, molecular weight **(B)**, and branching density **(C)** of ethylene oligomers produced with **Ni1**-**Ni3** relative to temperature at 30–70°C.

**FIGURE 3:**
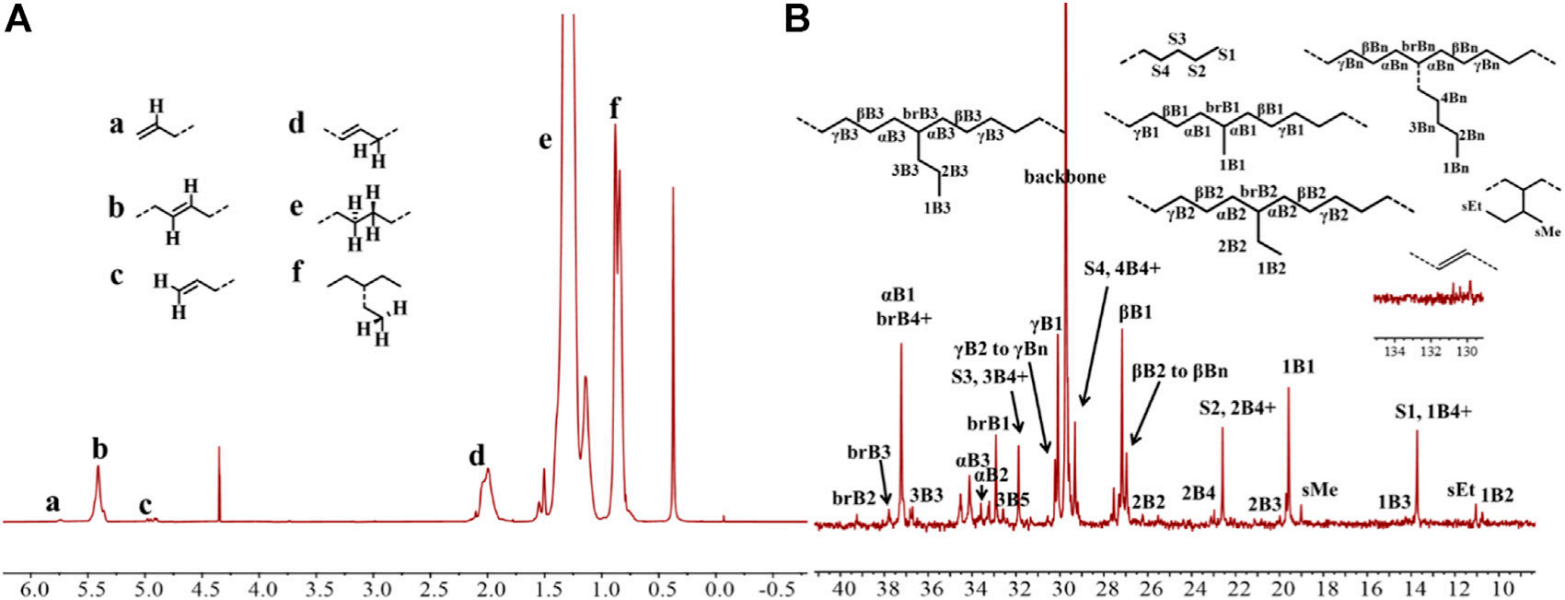
The ^1^H **(A)** and ^13^C **(B)** NMR spectral analyses of the hyperbranched ethylene oligomer obtained with **Ni2** at 70°C. Assignments are numbered based on ref. Cotts et al., 2000; Randall, 1989; Galland et al., 1999. Supplementary Figures S1−S4 are assigned to the chain ends. xBy delineates the branches, where x is the carbon, beginning with 1 at the methyl end and y is the branch length. brBy denotes the methine groups for branches with different lengths.

Upon activation by 200 eq. of Et_2_AlCl, the Ni(II) complexes demonstrated extremely high ethylene oligomerization activity (level of 10^6^–10^7^ g mol^−1^ h^−1^) and yielded low molecular weight (ca. 0.5–1.8 kg/mol) ethylene waxes with high branching densities (86–117/1000°C) (Table 1). As the reaction temperature increased, the catalytic activities of all the complexes in this system gradually decreased (Figure 2A). This is probably due to the decrease of ethylene solubility in toluene as the reaction temperature increases. A faster catalyst deactivation at higher temperatures may also play an important role. The molecular weight of the resulting ethylene oligomers reduced (Figure 2B), whereas the branching density elevated (Figure 2C) when the reaction temperatures were increased for all these complexes. Interestingly, compared to the benzocyclopentyl complex **Ni1**, the benzocyclohexyl complex **Ni2** produced higher molecular weight ethylene oligomers with lower branching densities (Figures 2B,C), which is caused by the fact that the cyclohexyl group provides a larger steric hindrance than the cyclopentyl group, since a larger steric hindrance in the α-diimine nickel system helps to obtain higher molecular weight polymers (Gong et al., 2019; Hai et al., 2021; Lu et al., 2021a; Zhao et al., 2021; Wang et al., 2022). Moreover, compared with the rigid planar naphthalene-based complex **Ni3**, the flexible stereoscopic benzocyclohexyl complex **Ni2** yielded higher molecular weight ethylene oligomers with similar branching density (Figures 2B,C). This may also be due to the greater steric hindrance of the cyclohexyl group over the phenyl substituent. In terms of catalytic activity, complex **Ni2** is less active than **Ni1** and **Ni3** at low temperatures while the opposite trend is observed at high temperatures (Figure 2A). This is most likely due to the greater thermal stability of the bulkier nickel complex **Ni2** at high temperatures although it is not conducive to the coordination and insertion of ethylene molecules. The above discussion indicates that the introduction of a flexible stereoscopic cyclohexyl group can improve the molecular weight and thermal stability of the catalytic system. The microstructure of a representative ethylene oligomer (Table 1, entry 6) was revealed using ^1^H and ^13^C NMR analyses (Figure 3). The resonance assignments for ^1^H NMR spectrum of the ethylene oligomer reveal the existence of a high amount of terminal methyl groups, a minor quantity of terminal double bonds, a major quantity of internal double bonds and a C=C-C*H*_2_ group (Figure 3A). Furthermore, the ^13^C NMR spectra reveal the existence of a branch-on-branch structure, a double bond and the branches with different chain lengths (ethyl, methyl, *n*-propyl, etc.) (Figure 3B). The chain end groups and methyl branches were the most common among the branches, and the presence of *sec*-butyl groups indicated that the ethylene oligomer possessed hyperbranched structures (Cotts et al., 2000).

**TABLE 1:** Ni(II) catalysts for ethylene oligomerization^a^.

Entry	Precatalyst	*T*/^°^C	Yield/g	Activity^b^	*M*_n (NMR)_ ^c^	*B* ^d^
1	**Ni1**	30	2.31	13.86	726	95
2	**Ni1**	50	1.53	9.18	543	105
3	**Ni1**	70	0.85	5.10	493	117
4	**Ni2**	30	1.59	9.54	1802	86
5	**Ni2**	50	1.58	9.48	1072	100
6	**Ni2**	70	1.08	6.48	792	102
7	**Ni3**	30	2.07	12.42	737	86
8	**Ni3**	50	1.46	8.76	645	99
9	**Ni3**	70	0.96	5.76	591	104

a Conditions: 1 μmol Ni(II) complexes, 200 eq. Et_2_AlCl, 20 ml toluene, 1 ml CH_2_Cl_2_, 10 min polymerization time, 6 atm.

b Activity = 10^6^ g/(mol Ni·h).

c *M*_n_ (g mol^−1^) estimated from ^1^H NMR, peak intensities.

d *B* is the number of branches per 1000 carbons, B = 1000 × 2(I_CH3_)/3(I_CH2+CH_ + I_CH3_), including saturated end groups.

### Pd(II) complexes catalyzed ethylene (co)oligomerization

**FIGURE 4:**
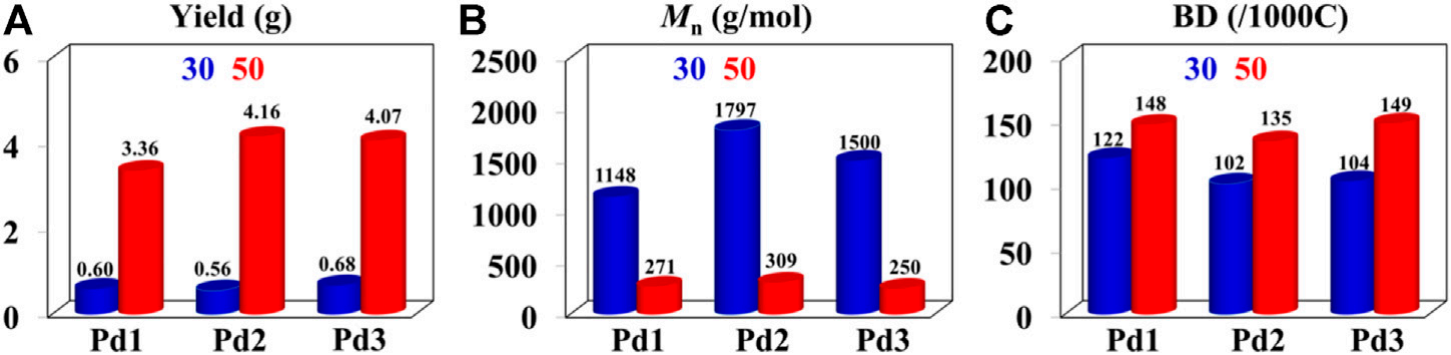
Comparisons on yield **(A)**, molecular weight **(B)**, and branching density **(C)** of ethylene oligomers produced by **Pd1**-**Pd3** at 30°C (bule) and 50°C (red).

**FIGURE 5:**
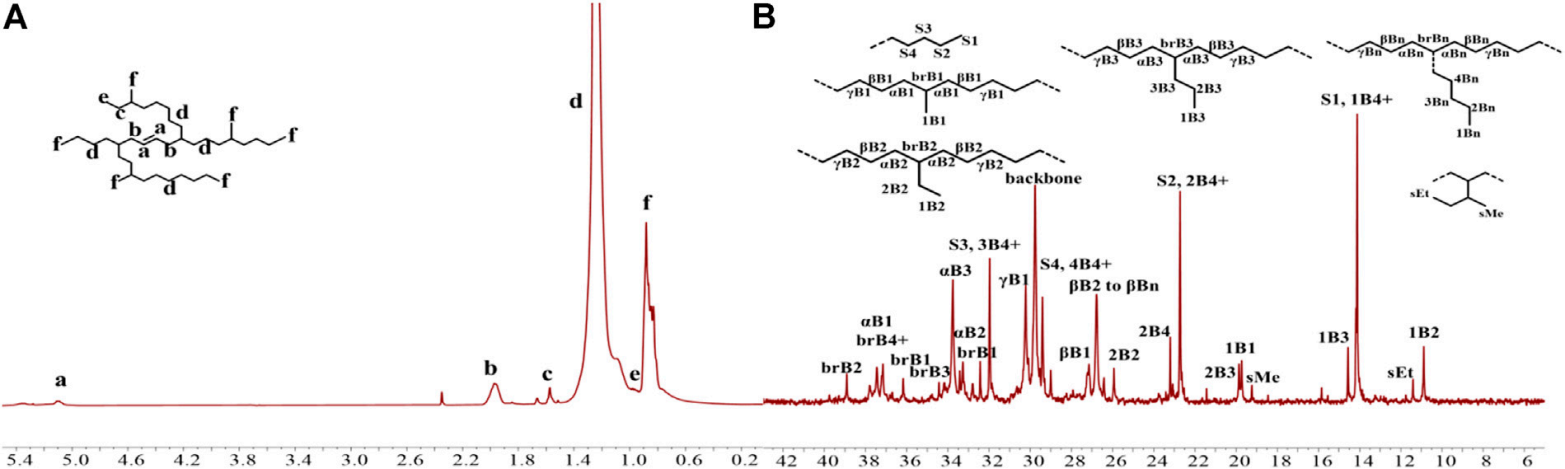
The ^1^H **(A)** and ^13^C **(B)** NMR spectral analyses of a hyperbranched ethylene oligomer obtained with **Pd1** at 30°C. Assignments are numbered based on ref. Cotts et al., 2000; Randall, 1989; Galland et al., 1999. Supplementary Figures S1−S4 are assigned to the chain ends. xBy delineates the branches, where x is the carbon, beginning with 1 at the methyl end and y is the branch length. brBy denotes the methine groups for branches with different lengths.

The iminopyridine Pd(II) complexes were also used for the ethylene oligomerization process, with activation by 2 equiv. of sodium tetrakis (3,5-bis(trifluoromethyl)phenyl)borate (NaBArF). All the Pd(II) complexes demonstrated moderate catalytic activity and produced colorless oil with low density. Notably, raising the temperature significantly increased the catalytic activity, whereas the molecular weights of the obtained ethylene oligomers significantly decreased. These results indicate that a high temperature favors chain transfer over chain propagation, and improves the rate of ethylene insertion (Figure 4). The elevated temperatures could lead to ethylene oligomers with significantly higher branching densities, which was not in line with the similarly structured α-diimine Pd(II) system, where the branching density of the obtained polyethylene was generally independent of temperature variations (Lu et al., 2022; Yan et al., 2022b). This may be attributed to the markedly lower molecular weights of the ethylene oligomers obtained at higher temperatures, resulting in a large amount of end groups. Interestingly, compared to the benzocyclopentyl complex **Pd1**, the benzocyclohexyl complex **Pd2** produced ethylene oligomers with higher molecular weights and lower branching densities (Figures 4B,C), similar to the corresponding nickel system. Moreover, compared to the rigid planar naphthalene-based complex **Pd3**, the flexible stereoscopic benzocyclohexyl complex **Pd2** yielded higher molecular weight ethylene oligomers with lower branching density. In terms of catalytic activity, complex **Pd2** is less active than **Pd1** and **Pd3** at 30°C while the opposite is observed at 50°C (Figure 4A). This is most likely due to the good thermal stability of the bulky palladium complex **Pd2** at high temperatures, similar to the corresponding nickel system. The above discussion indicates that the introduction of a flexible stereoscopic cyclohexyl group can also improve the thermal stability of the palladium system and the molecular weight of the resulting ethylene oligomers. The microstructure of a representative ethylene oligomer (entry 1, Table 2) were determined by ^1^H and ^13^C NMR analyses (Figure 5). The resonance assignments of ^1^H NMR spectrum of the ethylene oligomer reveal a high amount of terminal methyl groups, a C=C-C*H*_2_ group and an internal double bond (Figure 5A). Furthermore, the ^13^C NMR spectra reveal the existence of a branch-on-branch structure, an internal double bond, and the branches of different chain lengths (ethyl, methyl, *n*-propyl, etc.) (Figure 5B). The chain end groups and long-chain branches were the most common among the branches, and the presence of *sec*-butyl groups indicated that the ethylene oligomer possessed hyperbranched structures (Cotts et al., 2000).

**TABLE 2:** Pd(II) catalysts for ethylene oligomerization^a^.

Entry	Precatalyst	*T* (^°^C)	Yield (g)	Activity^b^	*M*_n (NMR)_ ^c^	*B* ^d^
1	**Pd1**	30	0.60	2.00	1148	122
2	**Pd1**	50	3.36	11.20	271	148
3	**Pd2**	30	0.56	1.87	1797	102
4	**Pd2**	50	4.16	13.87	309	135
5	**Pd3**	30	0.68	2.27	1500	104
6	**Pd3**	50	4.07	13.57	250	149

a Reaction conditions: 10 μmol Pd catalyst, 2.0 equiv. NaBArF, 4 atm Ethylene, 40 ml DCM, 3 h polymerization time.

b Activity is expressed as 10^4^ g mol^−1^ h^−1^.

c *M*_n_ (g mol^−1^) estimated from ^1^H NMR, peak intensities.

d B is the number of branches per 1000 carbons, B = 1000 × 2(I_CH3_)/3(I_CH2+CH_ + I_CH3_), including saturated end groups.

**FIGURE 6:**
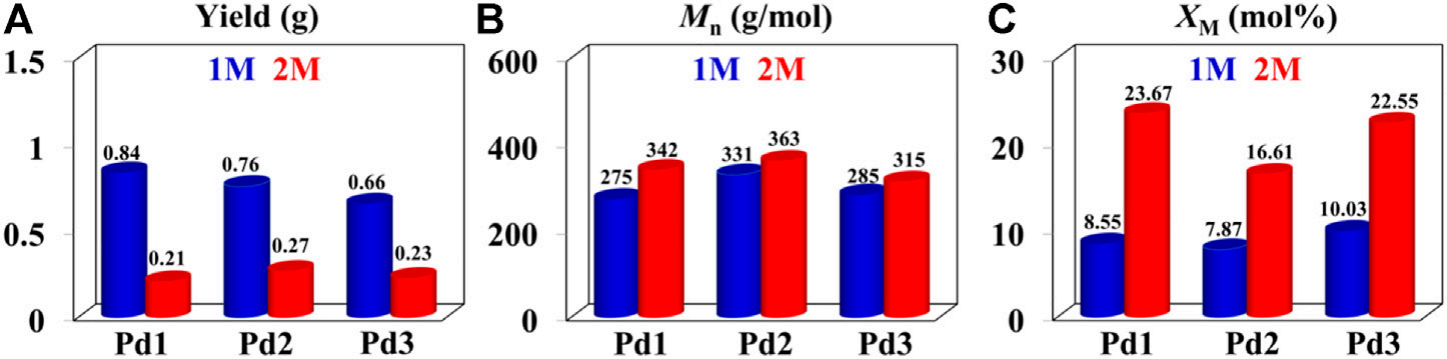
Comparisons on yield **(A)**, molecular weight **(B)** and incorporation ratio **(C)** of ethylene-MA co-oligomers generated with catalysts **Pd1**-**Pd3** at 1 M (bule) and 2 M (red).

**FIGURE 7:**
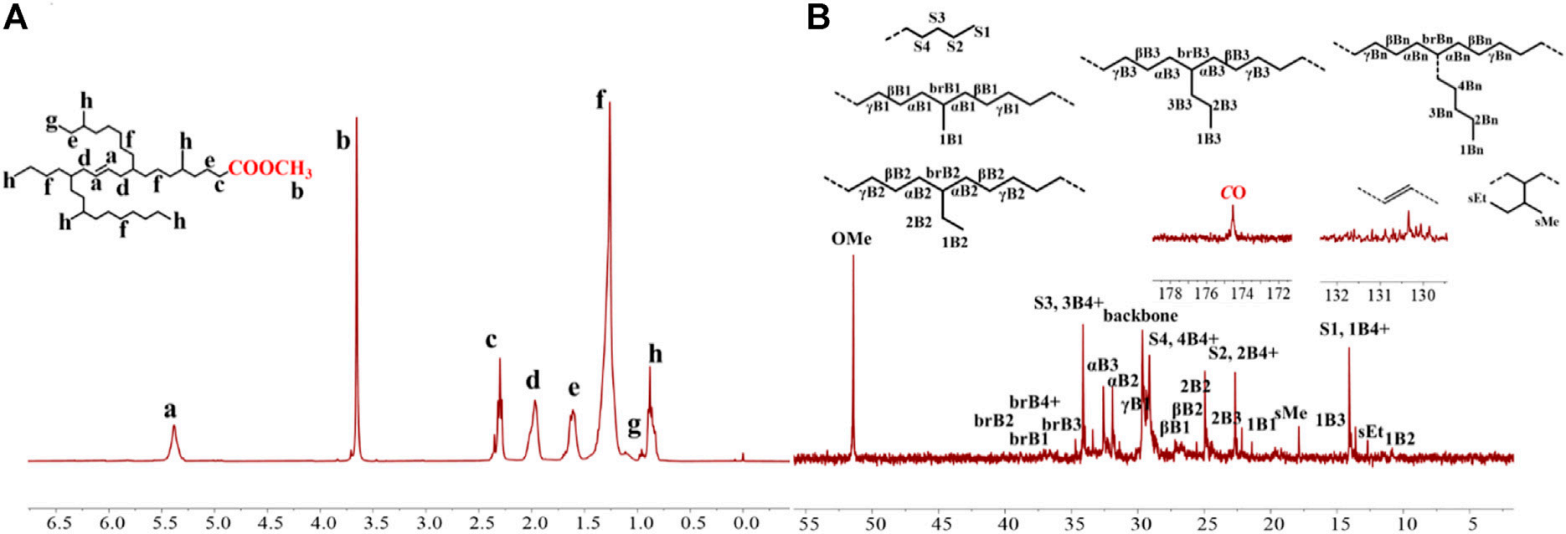
The ^1^H **(A)** and ^13^C **(B)** NMR spectral analyses of hyperbranched ethylene-MA co-oligomers obtained with **Pd1** at 1 M MA. Assignments are numbered based on ref. Cotts et al., 2000; Randall, 1989; Galland et al., 1999. Supplementary Figures S1−S4 are assigned to the chain ends. xBy delineates the branches, where x is the carbon, beginning with 1 at the methyl end and y is the branch length. brBy denotes the methine groups for branches with different lengths.

The iminopyridyl Pd(II) catalysts have been shown to be highly effective for copolymerizing olefins and polar monomers, resulting in the production of polar functionalized polyolefins with tunable molecular weights and high incorporation ratios. (Dai and Li, 2020; Ge et al., 2021; Li and Dai, 2021; Lu et al., 2021b; Peng et al., 2021; Fan et al., 2022a; Fan et al., 2022b; Yan et al., 2022a). In the present research, polar functionalized ethylene/MA co-oligomers with low molecular weights and very high incorporation ratios (up to 23.67 mol%) were yielded using **Pd1-Pd3** (Table 3). These Pd(II) complexes demonstrated significantly lower co-oligomerization activities (10^3^ g mol^−1^ h^−1^) than their homo-oligomerization activities, which might be attributed to the inhibiting effects of the COOMe groups. Upon doubling the MA concentrations, the co-oligomerization activities reduced significantly, whereas the incorporation ratios were markedly increased. Compared to the benzocyclopentyl complex **Pd1**, the benzocyclohexyl complex **Pd2** generated higher molecular weight ethylene-MA co-oligomers with lower branching densities and lower incorporation ratios (Figures 6B,C). Moreover, compared with the rigid planar naphthalene-based complex **Pd3**, the flexible stereoscopic benzocyclohexyl complex **Pd2** yielded higher molecular weight ethylene-MA co-oligomers with higher catalytic activities and lower incorporation ratios (Figures 6A–C). This is most likely due to the fact that the larger steric hindrance is not conducive to the coordination insertion of polar monomers, which will result in a higher catalytic activity and a higher molecular weight as well as a lower insertion ratio. The ethylene/MA co-oligomers produced with these Pd(II) catalysts from 2M MA solutions contain >1 polar functionalized group in each chain. The microstructure of a representative ethylene/MA co-oligomer (entry 1, Table 3) was elucidated by ^1^H and ^13^C NMR analyses (Figure 7). The assignments of the ^1^H NMR resonances of ethylene/MA co-oligomer reveal a high amount of terminal methyl groups, a C=C-C*H*_2_-group, a methoxyl group (OC*H*_3_), a C*H*_2_CH_2_COOMe, a C*H*_2_COOMe and an internal double bond (Figure 7A). Furthermore, the corresponding ^13^C NMR spectra reveal the existence of different branches with different chain lengths (ethyl, methyl, *n*-propyl, etc.), a branch-on-branch structure, an ester carbonyl group, an O*C*H_3_ group and an internal double bond (Figure 7B). The long-chain branches and those carrying chain end ester groups accounted for the majority of all chain branches and the hyperbranching was also detected based on the presence of *sec*-butyl groups (Cotts et al., 2000). The ester groups in the hyperbranched ethylene-MA co-oligomers are predominately incorporated at the branching end (Fan et al., 2022a; Fan et al., 2022b; Yan et al., 2022a).

**TABLE 3:** Co-oligomerization of methyl acrylate and ethylene with Pd(II) catalysts^a^.

Entry	Precatalyst	[MA] (mol L^−1^)	Yield (g)	Activity ^b^	*X* (mol%)^c^	*M*_n__(NMR)_ ^d^	*B* ^e^
1	**Pd1**	1	0.84	3.50	8.55	275	145
2	**Pd1**	2	0.21	0.88	23.67	342	159
3	**Pd2**	1	0.76	3.17	7.87	331	129
4	**Pd2**	2	0.27	1.13	16.61	363	137
5	**Pd3**	1	0.66	2.75	10.03	285	143
6	**Pd3**	2	0.23	0.96	22.55	315	157

a General conditions: 20 μmol Pd catalyst, 2.0 equiv. NaBArF, 4 atm ethylene, 12 h polymerization time, 20 ml total volume of CH_2_Cl_2_ and MA, 30°C polymerization temperature.

b Activity expressed as 10^3^ g mol^−1^ h^−1^.

c *X* = MA, incorporation.

d *M*_n_ (g mol^−1^) estimated from ^1^H NMR, peak intensities.

e B is the number of branches per 1000 carbons. The branches ending with functional groups are included as the total branches.

## Conclusion

A series of dibenzhydryl iminopyridyl ligands with benzocycloalkyl and naphthyl moieties and the corresponding Ni(II) and Pd(II) complexes were synthesized in excellent yields and characterized by NMR and mass spectrometries and elemental analyses. All the Ni(II) and Pd(II) complexes were employed as catalysts in ethylene oligomerization and ethylene/MA-co-oligomerization. In the Ni(II)-catalyzed ethylene oligomerizations, **Ni1-Ni3** exhibited very high catalytic activities (up to 13.86 × 10^6^ g mol^−1^ h^−1^) and produced highly branched (86-117/1000 C) ethylene oligomers with low molecular weights (493–1802 g/mol). In the Pd(II)-catalyzed ethylene oligomerizations, **Pd1-Pd3** exhibited moderate catalytic activities (1.87–13.87 × 10^4^ g mol^−1^ h^−1^) and yielded highly branched (102-149/1000 C) ethylene oligomers with low molecular weights (250–1797 g/mol). For the ethylene-MA co-oligomerizations, polar functionalized ethylene/MA co-oligomers with low molecular weights and very high incorporation ratios (up to 23.67 mol%) were produced using complexes **Pd1-Pd3**. Moreover, the flexible stereoscopic benzocyclohexyl complexes yielded the highest molecular weight ethylene oligomers or E-MA co-oligomers among these complexes. The reaction temperature, catalyst ligand structure and metal type have all significant effects on the ethylene (co) oligomerization with respect to catalytic activity, molecular weight and oligomer topology. Notably, all the produced ethylene oligomers and E-MA co-oligomers were demonstrated to contain hyperbranched microstructures with different topologies.

## Data Availability

The original contributions presented in the study are included in the article/Supplementary Material, further inquiries can be directed to the corresponding author.

## References

[B1] Albahily K. Gambarotta S. Duchateau R. (2011). Ethylene oligomerization promoted by a silylated-SNS chromium system. Organometallics 30, 4655–4664. 10.1021/om200505a

[B2] Alzamly A. Gambarotta S. Korobkov I. (2013). Synthesis, structures, and ethylene oligomerization activity of bis(phosphanylamine)pyridine chromium/aluminate complexes. Organometallics 32, 7107–7115. 10.1021/om4008289

[B3] Bianchini C. Giambastiani G. Luconi L. Meli A. (2010). Olefin oligomerization, homopolymerization and copolymerization by late transition metals supported by (imino)pyridine ligands. Coord. Chem. Rev. 254, 431–455. 10.1016/j.ccr.2009.07.013

[B4] Chen Z. Allen K. E. White P. S. Daugulis O. Brookhart M. (2016). Synthesis of branched polyEthylene with “half-sandwich” pyridine-imine nickel complexes. Organometallics 35, 1756–1760. 10.1021/acs.organomet.6b00165

[B5] Chen X. Gao J. Liao H. Gao H. Wu Q. (2018). Synthesis, characterization, and catalytic Ethylene oligomerization of pyridine-imine palladium complexes. Chin. J. Polym. Sci. 36, 176–184. 10.1007/s10118-018-2052-8

[B6] Cotts P. M. Guan Z. McCord E. McLain S. (2000). Novel branching topology in polyethylenes as revealed by light scattering and ^13^C NMR. Macromolecules 33, 6945–6952. 10.1021/ma000926r

[B7] Cruz T. F. C. Figueira C. A. Veiros L. F. Gomes P. T. (2021). Benzylnickel(II) complexes of 2-iminopyrrolyl chelating ligands: Synthesis, structure, and catalytic oligo-/polymerization of ethylene to hyperbranched PolyEthylene. Organometallics 40, 2594–2609. 10.1021/acs.organomet.1c00297

[B8] Dai S. Li S. (2020). Effect of aryl orientation on olefin polymerization in iminopyridyl catalytic system. Polymer 200, 122607. 10.1016/j.polymer.2020.122607

[B9] Dai S. Sui X. Chen C. (2016). Synthesis of high molecular weight polyEthylene using iminopyridyl nickel catalysts. Chem. Commun. 52, 9113–9116. 10.1039/c6cc00457a 27001556

[B10] D'Auria I. Milione S. Caruso T. Balducci G. Pellecchia C. (2017). Synthesis of hyperbranched low molecular weight polyEthylene oils by an iminopyridine nickel(II) Catalyst. Polym. Chem. 8, 6443–6454. 10.1039/c7py01215b

[B11] Falivene L. Wiedemann T. Göttker-Schnetmann I. Caporaso L. Cavallo L. Mecking S. (2018). Control of chain walking by weak neighboring group interactions in unsymmetrical catalysts. J. Am. Chem. Soc. 140, 1305–1312. 10.1021/jacs.7b08975 29261306

[B12] Fan H. Chang G. Bi H. Gui X. Wang H. Xu G. (2022a). Facile synthesis of hyperbranched ethylene oligomers and ethylene-methyl acrylate co-oligomers with different microscopic chain architectures. ACS Polym. Au 2, 88–96. 10.1021/acspolymersau.1c00039 PMC9954315 36855342

[B13] Fan H. Xu G. Wang H. Dai S. (2022b). Direct synthesis of hyperbranched ethene oligomers and ethene-MA co-oligomers using iminopyridyl systems with weak neighboring group interactions. J. Polym. Sci. 60, 1944–1953. 10.1002/pol.20220047

[B14] Galland G. B. De Souza R. F. Mauler R. S. Nunes F. F. (1999). ^13^C NMR determination of the composition of linear low-density polyethylene obtained with [η^3^-Methallyl-nickel-diimine]PF_6_ complex. Macromolecules 32, 1620–1625. 10.1021/ma981669h

[B15] Ge Y. Li S. Wang H. Dai S. (2021). Synthesis of branched Polyethylene and ethylene-MA copolymers using unsymmetrical iminopyridyl nickel and palladium complexes. Organometallics 40, 3033–3041. 10.1021/acs.organomet.1c00388

[B16] Gong Y. Li S. Gong Q. Zhang S. Liu B. Dai S. (2019). Systematic investigations of ligand steric effects on α-diimine nickel catalyzed olefin polymerization and copolymerization. Organometallics 38, 2919–2926. 10.1021/acs.organomet.9b00267

[B17] Guo L. Kong W. Xu Y. Yang Y. Ma R. Cong L. (2018). Large-scale synthesis of novel sterically hindered acenaphthene-based α-diimine ligands and their application in coordination chemistry. J. Organomet. Chem. 859, 58–67. 10.1016/j.jorganchem.2018.01.055

[B18] Guo L. Liu W. Li K. Sun M. Sun W. Zhao L. (2019). Synthesis of functional and hyperbranched Ethylene oligomers using unsymmetrical α-diimine palladium catalysts. Eur. Polym. J. 115, 185–192. 10.1016/j.eurpolymj.2019.03.030

[B19] Guo L. Li S. Ji M. Sun W. Liu W. Li G. (2019). Monoligated vs bisligated effect in iminopyridyl nickel catalyzed ethylene polymerization. Organometallics 38, 2800–2806. 10.1021/acs.organomet.9b00325

[B20] Hai Z. Lu Z. Li S. Cao Z.-Y. Dai S. (2021). The synergistic effect of rigid and flexible substituents on insertion polymerization with α-diimine nickel and palladium catalysts. Polym. Chem. 12, 4643–4653. 10.1039/d1py00812a

[B21] Hameury S. de Frémont P. Breuil P.-A. R. Olivier-Bourbigou H. Braunstein P. (2015). Bis(ether-functionalized NHC) nickel(II) complexes, trans to cis isomerization triggered by water coordination, and catalytic ethylene oligomerization. Organometallics 34, 2183–2201. 10.1021/om5008506

[B22] Huang F. Sun Z. Du S. Yue E. Ba J. Hu X. (2015). Ring-tension adjusted Ethylene polymerization by aryliminocycloheptapyridyl nickel complexes. Dalton Trans. 44, 14281–14292. 10.1039/c5dt01831e 26197778

[B23] Huang C. Zhang Y. Liang T. Zhao Z. Hu X. Sun W. (2016). Rigid geometry 8-arylimino-7, 7-dimethyl-5, 6-dihydroquinolyl nickel bromides: single-site active species towards ethylene polymerization. New J. Chem. 40, 9329–9336. 10.1039/c6nj02464e

[B24] Junges F. Kuhn M. C. A. dos Santos A. H. D. P. Rabello C. R. K. Thomas C. M. Carpentier J.-F. (2007). Chromium catalysts based on tridentate pyrazolyl ligands for ethylene oligomerization. Organometallics 26, 4010–4014. 10.1021/om070215i

[B25] Laine T. V. Lappalainen K. Liimatta J. Aitola E. Lofgren B. Leskela M. (1999). Polymerization of ethylene with new diimine complexes of late transition metals. Macromol. Rapid Commun. 20, 487–491. 10.1002/(sici)1521-3927(19990901)20:9<487::aid-marc487>3.0.co;2-g

[B26] Laine T. V. Piironen U. Lappalainen K. Klinga M. Aitola E. Leskela M. (2000). Pyridinylimine-based nickel(II) and palladium(II) complexes: preparation, structural characterization and use as alkene polymerization catalysts. J. Org. Chem. 606, 112–124. 10.1016/s0022-328x(00)00291-6

[B27] Li S. Dai S. (2021). Highly efficient incorporation of polar comonomers in copolymerizations with Ethylene using iminopyridyl palladium system. J. Catal. 393, 51–59. 10.1016/j.jcat.2020.11.015

[B28] Li S. Lu Z. Fan W. Dai S. (2021). Efficient incorporation of a polar comonomer for direct synthesis of hyperbranched polar functional Ethylene oligomers. New J. Chem. 45, 4024–4031. 10.1039/d0nj05857b

[B29] Liu S. Zhang Y. Han Y. Feng G. Gao F. Wang H. (2017). Selective ethylene oligomerization with chromium-based metal-organic framework MIL-100 evacuated under different temperatures. Organometallics 36, 632–638. 10.1021/acs.organomet.6b00834

[B30] Lu Z. Chang G. Wang H. Jing K. Dai S. (2021). A dual steric enhancement strategy in α-diimine nickel and palladium catalysts for ethylene polymerization and copolymerization. Organometallics 41, 124–132. 10.1021/acs.organomet.1c00568

[B31] Lu Z. Wang H. Li S. K. Dai S. Y. (2021). Direct synthesis of various polar functionalized polypropylene materials with tunable molecular weights and high incorporation ratios. Polym. Chem. 12, 5495–5504. 10.1039/d1py01064f

[B32] Lu W. Wang H. Fan W. Dai S. (2022). Exploring the relationship between polyethylene microstructure and spatial structure of α-diimine Pd(II) catalysts via A Hybrid steric strategy. Inorg. Chem. 61, 6799–6806. 10.1021/acs.inorgchem.1c03969 35476412

[B33] Luo W. Li A. Liu S. Ye H. Li Z. (2016). 2-Benzimidazol-6-pyrazol-pyridine chromium(III) trichlorides: Synthesis, characterization, and application for ethylene oligomerization and polymerization. Organometallics 35, 3045–3050. 10.1021/acs.organomet.6b00573

[B34] Mecking S. Schnitte M. (2020). Neutral nickel(II) catalysts: From hyperbranched oligomers to nanocrystal-based materials. Acc. Chem. Res. 53, 2738–2752. 10.1021/acs.accounts.0c00540 33094994

[B35] Meduri A. Montini T. Ragaini F. Fornasiero P. Zangrando E. Milani B. (2013). Palladium-catalyzed ethylene/methyl acrylate cooligomerization: Effect of a new nonsymmetric α-diimine. ChemCatChem 5, 1170–1183. 10.1002/cctc.201200520

[B36] Meneghetti S. P. Lutz P. J. Kress J. (1999). Oligomerization of olefins catalyzed by new cationic palladium(II) complexes containing an unsymmetrical α-diimine ligand. Organometallics 18, 2734–2737. 10.1021/om990165k

[B37] Mukherjee S. Patel B. A. Bhaduri S. (2009). Selective ethylene oligomerization with nickel oxime complexes. Organometallics 28, 3074–3078. 10.1021/om900080h

[B38] Nifant’ev I. E. Vinogradov A. A. Vinogradov A. A. Roznyatovsky V. A. Grishin Y. K. Ivanyuk A. V. (2018). 5, 6-Dihydrodibenzo [c, e][1, 2] azaphosphinine-based PNP ligands, Cr (0) coordination, and Cr (III) precatalysts for ethylene oligomerization. Organometallics 37, 2660–2664.

[B39] Olivier-Bourbigou H. Breuil P. A. R. Magna L. Michel T. Espada Pastor M. F. Delcroix D. (2020). Nickel catalyzed olefin oligomerization and dimerization. Chem. Rev. 120, 7919–7983. 10.1021/acs.chemrev.0c00076 32786672

[B40] Peng H. Li S. Li G. Dai S. Ji M. Liu Z. (2021). Rotation-restricted strategy to synthesize high molecular weight polyethylene using iminopyridyl nickel and palladium catalyst. Appl. Organomet. Chem. 35, e6140. 10.1002/aoc.6140

[B41] Randall J. C. (1989). A review of high-resolution liquid carbon-13 nuclear magnetic resonance characterizations of ethylene-based polymer. J. Macromol. Sci. 29, 201–317. 10.1080/07366578908055172

[B42] Rosa V. Avilés T. Aullon G. Covelo B. Lodeiro C. (2008). A new bis (1-naphthylimino) acenaphthene compound and its Pd(II) and Zn(II) complexes: synthesis, characterization, solid-state structures and density functional theory studies on the syn and anti isomers. Inorg. Chem. 47, 7734–7744. 10.1021/ic800790u 18671386

[B43] Saki Z. D'Auria I. Dall'Anese A. Milani B. Pellecchia C. (2020). Copolymerization of ethylene and methyl acrylate by pyridylimino Ni(II) catalysts affording hyperbranched poly(ethylene-co-methyl acrylate)s with tunable structures of the ester groups. Macromolecules 53, 9294–9305. 10.1021/acs.macromol.0c01703

[B44] Stephenson C. J. McInnis J. P. Chen C. Weberski M. P. Motta A. Delferro M. (2014). Ni(II) phenoxyiminato olefin polymerization catalysis: Striking coordinative modulation of hyperbranched polymer microstructure and stability by a proximate sulfonyl group. ACS Catal. 4, 999–1003. 10.1021/cs500114b

[B45] Sydora O. L. (2019). Selective ethylene oligomerization. Organometallics 38, 997–1010. 10.1021/acs.organomet.8b00799

[B46] Tomov A. K. Chirinos J. J. Long R. J. Gibson V. C. Elsegood M. R. J. (2006). An unprecedented α-olefin distribution arising from a homogeneous ethylene oligomerization catalyst. J. Am. Chem. Soc. 128, 7704–7705. 10.1021/ja0615369 16771461

[B47] Wang H. Duan G. Fan H. Dai S. (2022). Second coordination sphere effect of benzothiophene substituents on chain transfer and chain walking in ethylene insertion polymerization. Polymer 245, 124707. 10.1016/j.polymer.2022.124707

[B48] Wiedemann T. Voit G. Tchernook A. Roesle P. Göttker-Schnetmann I. Mecking S. (2014). Monofunctional hyperbranched ethylene oligomers. J. Am. Chem. Soc. 136, 2078–2085. 10.1021/ja411945n 24450458

[B49] Xiang P. Ye Z. Subramanian R. (2011). Synthesis and characterization of low- and medium-molecular-weight hyperbranched polyEthylenes by chain walking Ethylene polymerization with Pd-diimine catalysts. Polymer 52, 5027–5039. 10.1016/j.polymer.2011.09.010

[B50] Yan Z. Li S. Dai S. (2021). Synthesis and characterization of hyperbranched polar functionalized olefin oligomers. Chin. J. Synth. Chem. 29, 1033–1044. 10.15952/j.cnki.cjsc.1005-1511.21166

[B51] Yan Z. Bi H. Ding B. Wang H. Xu G. Dai S. (2022). A rigid-flexible double-layer steric strategy for ethylene (Co) oligomerization with pyridine-imine Ni (II) and Pd (II) complexes. New J. Chem. 41, 8669–8678. 10.1039/D2NJ00183G

[B52] Yan Z. Xu G. Wang H. Dai S. (2022). Synthesis of thermoplastic polyethylene elastomers and ethylene–methyl acrylate copolymers using methylene-bridged binuclear bulky dibenzhydryl α-diimine Ni (II) and Pd (II) catalysts. Eur. Polym. J. 168, 111105. 10.1016/j.eurpolymj.2022.111105

[B53] Yang Y. Gurnham J. Liu B. Duchateau R. Gambarotta S. Korobkov I. (2014). Selective ethylene oligomerization with chromium complexes bearing pyridine-phosphine ligands: influence of ligand structure on catalytic behavior. Organometallics 33, 5749–5757. 10.1021/om5003683

[B54] Yeh B. Vicchio S. P. Chheda S. Zheng J. Schmid J. Löbbert L. (2021). Site densities, rates, and mechanism of stable Ni/UiO-66 ethylene oligomerization catalysts. J. Am. Chem. Soc. 143, 20274–20280. 10.1021/jacs.1c09320 34817993

[B55] Yue E. Xing Q. Zhang L. Shi Q. Cao X. Wang L. (2014). Synthesis and characterization of 2-(2-benzhydrylnaphthyliminomethyl) pyridylnickel halides: formation of branched polyethylene. Dalton Trans. 43, 3339–3346. 10.1039/c3dt53205d 24366281

[B56] Zhao Y. Li S. Fan W. Dai S. (2021). Reversion of the chain walking ability of α-diimine nickel and palladium catalysts with bulky diarylmethyl substituents. J. Organomet. Chem. 932, 121649. 10.1016/j.jorganchem.2020.121649

