# Supplementary material for: Ethylene (co) oligomerization using iminopyridyl Ni(II) and Pd(II) complexes bearing benzocycloalkyl moieties to access hyperbranched ethylene oligomers and ethylene-MA co-oligomers
Source: Front Chem. 2022 Aug 4;10:961426. doi: 10.3389/fchem.2022.961426 (PMC9386154; doi:10.3389/fchem.2022.961426)
Supplement: Supplementary file 1 [file DataSheet1.docx]

**Ethylene (Co)oligomerization Using Iminopyridyl Ni(II) and Pd(II) Complexes Bearing** **Benzocycloalkyl Moieties to Access Hyperbranched Ethylene Oligomers and Ethylene-MA Co-oligomers**

Beihang Ding^†^*^a,b^*, Guanru Chang^†^*^c^*, Zhengpeng Yan*^a^*, Shengyu Dai* *^a,b^*

*^a^Institutes of Physical Science and Information Technology, Key Laboratory of Structure and Functional Regulation of Hybrid Materials of Ministry of Education, Anhui University, Hefei, Anhui, 230601, China.*

*^b^School of Chemical and Environmental Engineering, Anhui Polytechnic University, Wuhu, Anhui 241000, China.*

*^c^School of Chemistry and Chemical Engineering, Key Laboratory of Inorganic Functional Material, Huangshan University, Huangshan, Anhui 245041, China*

^†^The first two authors are equal first authors.

**1. Experimental sections**

**1.1 General Considerations**

All chemicals were commercially sourced, except those whose synthesis is described. All experiments were carried out under a dry nitrogen atmosphere using standard Schlenk techniques or in a glove-box. Deuterated solvents used for NMR were dried and distilled prior to use. ^1^H and ^13^C NMR spectra were recorded by a JNM-ECZ600R or JNM-ECZ400R spectrometer at ambient temperature unless otherwise stated. The chemical shifts of the ^1^H and ^13^C NMR spectra were referenced to the residual solvent; Coupling constants are in Hz. Mass spectra were obtained by the Analytical Center of Anhui University. Elemental analysis was performed by the Analytical Center of Anhui University. X-ray Diffraction data were collected at 293(2) K on a Bruker Smart CCD area detector with graphite-monochromated Mo K^α^ radiation (λ = 0.71073 Å).

**1.2 Procedure for the Synthesis of Amines A1-A3.**

A mixture of Aromatic amine (10.0 mmol, 1.0 equiv.) and diphenylmethanol (20 mmol, 2.0 equiv.) was heated to 120 °C. A solution of anhydrous zinc chloride (5 mmol, 0.5 equiv.) in concentrated hydrochloric acid (1.0 equiv.) was added to the mixture (exothermic + intense bubbling), and the temperature was raised to 160 °C. After 30 min at 160 °C, the reaction mixture was cooled to room temperature and dissolved in CH_2_Cl_2_ (200 mL). The CH_2_Cl_2_ layer was washed with water (3 × 200 mL) and dried over anhydrous magnesium sulfate. The solution was concentrated to 20 mL. The product was crashed out with 200 ml methanol and washed with methanol (3 × 100 mL). The desired aniline was obtained as a white crystalline solid.

**A1** (4.19g , 90%,) ^1^H NMR (400 MHz, CDCl_3_) δ 7.25-7.06 (m, 12H, Ar-*H*), 7.00 (d, *J* = 6.8 Hz, 4H, Ar-*H*), 6.93 (d, *J* = 6.8 Hz, 4H, Ar-*H*), 6.01 (s, 1H, Ar-*H*), 5.38 (d, *J* = 4.5 Hz, 2H, -C*H*-), 3.30 (s, *br*, 2H, -N*H*_2_), 2.67 (dt, *J* = 12.3, 7.5 Hz, 4H, -C*H*_2_-), 2.09-1.96 (m, 2H, -C*H*_2_-). ^13^C NMR (101 MHz, CDCl_3_) δ 143.80, 142.79, 142.27, 138.49, 129.77, 129.53, 129.28, 129.22, 128.35, 128.01, 126.58, 126.35, 125.79, 54.02 (-*C*H-), 52.18 (-*C*H-), 31.78 (-*C*H_2_-), 29.80 (-*C*H_2_-), 24.41 (-*C*H_2_-). APCI-MS (m/z): calcd for C_35_H_32_N: 466.2535, Found, 466.2527, [M+H]^+^.

**A2** (3.98 g, 83%) ^1^H NMR (400 MHz, CDCl_3_) δ 7.13 - 6.95 (m, 12H, Ar-*H*), 6.88 (d, *J* = 6.6 Hz, 4H, Ar-*H*), 6.79 (d, *J* = 6.8 Hz, 4H, Ar-*H*), 5.91 (s, 1H, Ar-*H*), 5.43 (s, 1H, -C*H*-), 5.28 (s, 1H, -C*H*-), 3.30 (s, *br*, 2H, -N*H*_2_), 2.48 (t, *J* = 5.9 Hz, 2H, -C*H*_2_-), 2.34 (t, *J* = 6.1 Hz, 2H -C*H*_2_-), 1.71 - 1.62 (m, 2H -C*H*_2_-), 1.59 - 1.55 (m, 2H -C*H*_2_-). ^13^C NMR (101 MHz, CDCl_3_) δ 144.29, 142.69, 140.26, 134.23, 131.55, 129.50, 129.38, 129.32, 128.39, 128.05, 126.38, 125.77, 124.95, 121.93, 52.57 (-C*H*-), 52.43 (-C*H*-), 26.75 (-C*H*_2_-), 25.11 (-C*H*_2_-), 22.78 (-C*H*_2_-), 22.67 (-C*H*_2_-). APCI-MS (m/z): calcd for C_36_H_34_N: 480.2691, Found, 480.2681, [M+H]^+^.

**A3** (3.87 g, 81%) is known.^1^

**1.2 Procedure for the Synthesis of Ligands L1-L3.**

A round-bottom flask was charged with ZnCl_2_ (0.34 g, 2.5 mmol), 2-acetylpyridine (2.0 mmol), and CH_3_COOH (5 mL). Arylamines (2.0 mmol) was added, and the solution was heated to reflux for 4 h. After it was cooled to room temperature, the mixture was filtered to obtain a yellow solid, which was washed with diethyl ether (3 × 10 mL). This solid was placed in a round-bottom flask containing CH_2_Cl_2_ (100 mL). An aqueous solution (20 mL) of potassium oxalate monohydrate (0.4 g) was added to the stirred CH_2_Cl_2_ solution. After 1 h the layers were separated and the organic layer was washed with H_2_O (3 × 10 mL) and dried over MgSO_4_. The solvent was removed under vacuum to give a yellow solid.

**L1** (0.59 g, 69%). ^1^H NMR (400 MHz, CDCl_3_) δ 8.59 (d, *J* = 4.2 Hz, 1H, Ar-*H*), 8.26 (d, *J* = 7.9 Hz, 1H, Ar-*H*), 7.76 (t, *J* = 7.1 Hz, 1H, Ar-*H*), 7.33 (t, 1H, Ar-*H*), 7.25 - 7.01 (m, 14H, Ar-*H*), 6.95 (t, *J* = 6.6 Hz, 4H, Ar-*H*), 6.87 (d, *J* = 4.3 Hz, 2H, Ar-*H*), 6.33 (s, 1H, Ar-*H*), 5.47 (s, 1H, -C*H*-), 5.40 (s, 1H, -C*H*-), 2.74 - 2.64 (m, 2H, -C*H*_2_-), 2.62 - 2.53 (m, 1H, -C*H*_2_-), 2.46 (dt, *J* = 16.0, 7.9 Hz, 1H, -C*H*_2_-), 2.01 - 1.86 (m, 2H, -C*H*_2_-), 1.60 (s, 3H, Ar-C(C*H*_3_)=N). ^13^C NMR (101 MHz, CDCl_3_) δ 167.81 (Ar-*C*(CH_3_)=N), 156.38, 148.43, 143.81, 143.65, 143.58, 143.52, 143.06, 142.09, 136.25, 134.24, 131.81, 131.56, 129.59, 129.34, 129.21, 129.19, 129.08, 128.10, 128.03, 127.84, 125.98, 125.92, 125.74, 124.61, 121.21, 54.12 (-*C*H-), 52.12 (-*C*H-), 31.63 (-*C*H_2_-), 30.75 (-*C*H_2_-), 24.82 (-*C*H_2_-), 16.66 (Ar-C(*C*H_3_)=N). APCI-MS (m/z): calcd for C_42_H_37_N_2_: 569.2957, Found, 569.2940, [M+H]^+^.

**L2** (0.87 g, 75%). ^1^H NMR (400 MHz, CDCl_3_) δ 8.59 (d, *J* = 4.5 Hz, 1H, Ar-*H*), 8.28 (d, *J* = 7.9 Hz, 1H, Ar-*H*), 7.75 (t, *J* = 7.7 Hz, 1H, Ar-*H*), 7.34 - 7.29 (m, 1H, Ar-*H*), 7.23 - 7.03 (m, 12H, Ar-*H*), 6.98 (d, *J* = 7.1 Hz, 2H, Ar-*H*), 6.94 - 6.87 (m, 4H, Ar-*H*), 6.86 - 6.82 (m, 2H, Ar-*H*), 6.31 (s, 1H, Ar-*H*), 5.60 (s, 1H, -C*H*-), 5.28 (s, 1H, -C*H*-), 2.71 (dd, *J* = 17.1, 4.6 Hz, 1H, -C*H*_2_-), 2.50 (dd, *J* = 16.7, 8.9 Hz, 1H, -C*H*_2_-), 2.40 (d, *J* = 17.3 Hz, 1H, -C*H*_2_-), 2.12 (dd, *J* = 15.7, 9.9 Hz, 1H, -C*H*_2_-), 1.81 – 1.61 (m, 3Hz, 4H, -C*H*_2_-), 1.54 (s, 3H, Ar-C(C*H*_3_)=N). ^13^C NMR (101 MHz, CDCl_3_) δ 168.43(Ar-*C*(CH_3_)=N), 156.29, 148.50, 146.09, 144.16, 143.88, 143.60, 142.87, 136.51, 136.27, 133.93, 129.64, 129.50, 129.35, 129.23, 129.15, 129.01, 128.10, 128.05, 127.83, 125.99, 125.95, 125.88, 125.71, 124.83, 124.67, 121.16, 52.62 (-*C*H-), 52.09 (-*C*H-), 26.65 (-*C*H_2_-), 25.99 (-*C*H_2_-), 23.05 (-*C*H_2_-), 22.58 (-*C*H_2_-), 16.79 (Ar-C(*C*H_3_)=N). APCI-MS (m/z): calcd for C_43_H_39_N_2_: 583.3113, Found, 583.3096, [M+H]^+^.

**L3** (0.96 g, 83%) is known.^1^

**1.3 Procedure for the Synthesis of Nickel Complexes Ni1-Ni3.**

Complexes **Ni1-Ni3** were synthesized by the reaction of 1 equiv. of (DME)NiBr_2_ with the corresponding ligands in methylene chloride. The corresponding ligand (0.2 mmol) was added in 5 mL of methylene chloride in a Schlenk tube under a nitrogen atmosphere. (DME)NiBr_2_ (0.2 mmol, 62 mg) was added to the above solution. The resulting mixture was stirred at room temperature overnight. The solvent was evaporated under reduced pressure to afford a solid. The product was washed with 4 × 5 mL ether and dried under vacuum.

**Ni1** (0.148 g, 94%), MALDI-TOF-MS (m/z): calcd for C_42_H_36_BrN_2_Ni: 705.1415, Found, 705.1394, [M-Br]^+^. Elem. Anal. Calcd for C_42_H_36_Br_2_N_2_Ni: C, 64.08; H, 4.61; N, 3.56. Found: C, 64.17; H, 4.52; N, 3.61.

**Ni2** (0.154 g, 96%), MALDI-TOF-MS (m/z): calcd for C_43_H_38_BrN_2_Ni: 719.1572, Found, 719.1571, [M-Br]^+^. Elem. Anal. Calcd for C_43_H_38_Br_2_N_2_Ni: C, 64.45; H, 4.78; N, 3.50. Found: C, 64.57; H, 4.66; N, 3.62.

**Ni3** (0.141 g, 88%). Elem. Anal. Calcd for C_43_H_34_Br_2_N_2_Ni: C, 64.28; H, 4.30; N, 3.51. Found: C, 64.35; H, 4.41; N, 3.42.

**1.4 Procedure for the Synthesis of Palladium Complexes Pd1-Pd3.**

A mixture of the ligand (0.4 mmol), (COD)PdMeCl (106 mg, 0.4 mmol) in CH_2_Cl_2_ (10 mL) was stirred for 24 h at room temperature. During stirring, the color of the solution was deepening. At the end of the reaction, the solvent was partially evaporated under reduced pressure. The remaining mixture was diluted with Et_2_O (20 mL). The resulting yellow solid was collected by filtration, dried in vacuum. The single crystal can be obtained by diffusion from layering diethyl ether on to the CH_2_Cl_2_ solution at room temperature.

**Pd1** (0.26 g, 90%). ^1^H NMR (400 MHz, CDCl_3_) δ 9.27 (d, *J* = 4.7 Hz, 1H, Ar-*H*), 7.95 (t, *J* = 7.6 Hz, 1H, Ar-*H*), 7.71 - 7.66 (m, 1H, Ar-*H*), 7.41 (d, *J* = 7.9 Hz, 1H, Ar-*H*), 7.25 - 6.98 (m, 15H, Ar-*H*), 6.93 - 6.88 (m, 5H, Ar-*H*), 6.41 (s, 1H, Ar-*H*), 5.96 (s, 1H, -C*H*-), 5.47 (s, 1H, -C*H*-), 2.95 - 2.79 (m, 1H, -C*H*_2_-), 2.69 (t, *J* = 7.3 Hz, 2H, -C*H*_2_-), 2.41 - 2.27 (m, 1H, -C*H*_2_-), 2.09 - 1.88 (m, 2H, -C*H*_2_-), 1.15 (s, 3H, Ar-C(C*H*_3_)=N), 0.63 (s, 3H, Pd-CH_3_). ^13^C NMR (101 MHz, CDCl_3_) δ 175.73 (Ar-*C*(CH_3_)=N), 152.90, 149.59, 143.28, 142.97, 142.94, 142.32, 142.14, 139.55, 138.77, 138.19, 135.57, 134.20, 130.45, 129.66, 129.61, 129.32, 129.27, 128.72, 128.60, 128.52, 128.26, 126.65, 126.56, 126.48, 126.44, 124.54, 54.52 (-*C*H-), 52.19 (-*C*H-), 31.86 (-*C*H_2_-), 30.81 (-*C*H_2_-), 24.95 (-*C*H_2_-), 17.52 (Ar-C(*C*H_3_)=N), 0.78 (Pd-*C*H_3_). MALDI-TOF-MS (m/z): calcd for C_42_H_36_N_2_Pd: 674.1913, Found, 674.1898, [M-Cl-Me]^+^. Elem. Anal. Calcd for C_43_H_39_ClN_2_Pd: C, 71.17; H, 5.42; N, 3.86. Found: C, 71.24; H, 5.38; N, 3.68.

**Pd2** (0.27 g, 92%). ^1^H NMR (400 MHz, CDCl_3_) δ 9.27 (d, *J* = 4.9 Hz, 1H, Ar-*H*), 7.96 (t, *J* = 7.8 Hz, 1H, Ar-*H*), 7.69 (dd, *J* = 7.1, 5.6 Hz, 1H, Ar-*H*), 7.39 (d, *J* = 7.9 Hz, 1H, Ar-*H*), 7.28 - 7.18 (m, 4H, Ar-*H*), 7.15 - 7.08 (m, 4H, Ar-*H*), 7.03 (d, *J* = 4.4 Hz, 4H, Ar-*H*), 6.95 (d, *J* = 7.0 Hz, 2H, Ar-*H*), 6.89 - 6.81 (m, 6H, Ar-*H*), 6.39 (s, 1H, Ar-*H*), 5.91 (s, 1H, -C*H*-), 5.59 (s, 1H, -C*H*-), 2.89 (d, *J* = 16.8 Hz, 1H, -C*H*_2_-), 2.78 - 2.64 (m, 1H, -C*H*_2_-), 2.53 (dd, *J* = 15.4, 8.9 Hz, 1H, -C*H*_2_-), 1.95 (dd, *J* = 15.6, 7.4 Hz, 1H, -C*H*_2_-), 1.80 - 1.67 (m, 2H, -C*H*_2_-), 1.61 - 1.50 (m, 2H, -C*H*_2_-), 1.09 (s, 3H, Ar-C(C*H*_3_)=N), 0.60 (s, 3H, Pd-C*H*_3_). ^13^C NMR (101 MHz, CDCl_3_) δ 176.08 (Ar-*C*(CH_3_)=N), 152.83, 149.52, 143.83, 143.28, 142.17, 142.06, 141.49, 140.60, 138.82, 134.79, 132.46, 130.41, 129.72, 129.39, 129.22, 128.75, 128.55, 128.51, 128.45, 128.21, 126.70, 126.49, 126.42, 126.37, 124.55, 53.02 (-*C*H-), 52.12 (-*C*H-), 26.76 (-*C*H_2_-), 26.29 (-*C*H_2_-), 23.00 (-*C*H_2_-), 22.45 (-*C*H_2_-), 17.54 (Ar-C(*C*H_3_)=N), 0.94 (Pd-*C*H_3_). MALDI-TOF-MS (m/z): calcd for C_43_H_38_N_2_Pd: 688.2070, Found, 688.2080, [M-Cl-Me]^+^. Elem. Anal. Calcd for C_44_H_41_ClN_2_Pd: C, 71.45; H, 5.59; N, 3.79. Found: C, 71.33; H, 5.45; N, 3.79.

**Pd3** (0.27 g, 91%).^1^H NMR (400 MHz, CDCl3) δ 9.35 (d, *J* = 4.4 Hz, 1H, Ar-*H*), 8.04 - 7.95 (m, 2H, Ar-*H*), 7.74 (dd, *J* = 7.1, 5.6 Hz, 1H, Ar-*H*), 7.64 (dd, *J* = 6.6, 2.9 Hz, 1H, Ar-*H*), 7.46 (d, *J* = 7.9 Hz, 1H, Ar-*H*), 7.38 (dd, *J* = 6.4, 3.2 Hz, 2H, Ar-*H*), 7.26 - 7.02 (m, 14H, Ar-*H*), 6.92 (d, *J* = 7.9 Hz, 6H, Ar-*H*), 6.74 (s, 1H, Ar-*H*), 6.21 (s, 1H, -C*H*-), 6.19 (s, 1H, -C*H*-), 1.03 (s, 3H, Ar-C(C*H*_3_)=N), 0.56 (s, 3H, Pd-CH_3_). ^13^C NMR (101 MHz, CDCl_3_) δ 177.93 (Ar-*C*(CH_3_)=N), 152.70, 149.70, 143.69, 143.27, 141.77, 141.68, 139.39, 138.95, 138.90, 131.80, 131.03, 130.38, 129.72, 129.45, 129.25, 128.85, 128.75, 128.69, 128.45, 127.16, 126.90, 126.77, 126.74, 126.69, 126.65, 126.39, 125.30, 124.87, 123.23, 122.80, 53.38 (-*C*H-), 52.33 (-*C*H-), 18.56 (Ar-C(*C*H_3_)=N), 0.90 (Pd-*C*H_3_). Elem. Anal. Calcd for C_44_H_37_ClN_2_Pd: C, 71.84; H, 5.07; N, 3.81. Found: C, 71.67; H, 5.13; N, 3.74.

**1.5 A general procedure for the ethylene oligomerization using Ni complexes.**

In a typical experiment, a 350 mL stainless pressure reactor connected with a high pressure gas line was firstly dried at 90 °C under vacuum for at least 1 h. The reactor was then adjusted to the desired oligomerization temperature. 20 mL of toluene and the desired amount Et_2_AlCl was added to the reactor under N_2_ atmosphere, then the desired amount of catalyst in 1 mL of CH_2_Cl_2_ was injected into the oligomerization system via syringe. With a rapid stirring, the reactor was pressurized and maintained at 6 atm of ethylene. After 10 min, the pressure reactor was vented and the ethylene oligomers evaporated by a rotary evaporator and dried at 50 °C for at least 24 h under vacuum.

**1.6 A general procedure for the ethylene oligomerization using Pd complexes.**

In a typical experiment, a 350 mL stainless pressure reactor connected with a high pressure gas line was firstly dried at 90 °C under vacuum for at least 1 h. The reactor was then adjusted to the desired oligomerization temperature. 38 mL of DCM and desired amount NaBArF were added to the reactor, then the Pd catalyst in 2 mL of CH_2_Cl_2_ was injected into the oligomerization system via syringe subsequently. With a rapid stirring, the reactor was pressurized and maintained at 4 atm of ethylene. After 3 h, the pressure reactor was vented and the ethylene oligomers were dried under vacuum by a rotary evaporator.

**1.7 A general procedure for the co-oligomerization of MA with ethylene using Pd complexes.**

In a typical experiment, a 350 mL stainless pressure reactor connected with a high pressure gas line was firstly dried at 90 °C under vacuum for at least 1 h. The reactor was then adjusted to the desired oligomerization temperature. 18 mL of DCM with the desired amount NaBArF was added to the reactor, then the desired MA and Pd catalyst in 2 mL of CH_2_Cl_2_ was injected into the co-oligomerization system via syringe subsequently. With a rapid stirring, the reactor was pressurized and maintained at 4 atm of ethylene. After 12 h, the pressure reactor was vented and the ethylene-MA co-oligomers were dried under vacuum by a rotary evaporator.

**2. Spectra Data**

**2.1 ^1^H and ^13^C of the Synthetic Compounds.**

**Figure S1.** ^1^H NMR spectrum of **A1** in CDCl_3_.

**Figure S2.** ^13^C NMR spectrum of **A1** in CDCl_3_.

**Figure S3.** ^1^H NMR spectrum of **A2** in CDCl_3_.

**Figure S4.** ^13^C NMR spectrum of **A2** in CDCl_3_.

**Figure S5.** ^1^H NMR spectrum of **L1** in CDCl_3_.

**Figure S6.** ^13^C NMR spectrum of **L1** in CDCl_3_.

**Figure S7.** ^1^H NMR spectrum of **L2** in CDCl_3_.

**Figure S8.** ^13^C NMR spectrum of **L2** in CDCl_3_.

**Figure S9.** ^1^H NMR spectrum of **Pd1** in CDCl_3_.

**Figure S10.** ^13^C NMR spectrum of **Pd1** in CDCl_3_.

**Figure S11.** ^1^H NMR spectrum of **Pd2** in CDCl_3_.

**Figure S12.** ^13^C NMR spectrum of **Pd2** in CDCl_3_.

**Figure S13.** ^1^H NMR spectrum of **Pd3** in CDCl_3_.

**Figure S14.** ^13^C NMR spectrum of **Pd3** in CDCl_3_.

**2.2 MS of A1-A2 and L1-L2.**

**Figure S15.** APCI-MS of **A1**.

**Figure S16.** APCI-MS of **A2**.

**Figure S17.** APCI-MS of **L1**.

**Figure S18.** APCI-MS of **L2**.

**2.3 MS of Complexes Ni1-Ni2 and Pd1-Pd2.**

**Figure S19.** MALDI-TOF-MS of **Ni1**.

**Figure S20.** MALDI-TOF-MS of **Ni2**.

**Figure S21.** MALDI-TOF-MS of **Pd1**.

**Figure S22.** MALDI-TOF-MS of **Pd2**.

**2.4 ^1^H and ^13^C NMR of Representative Ethylene Oligomers and** **E-MA Co-oligomers.**

**Figure S23.** ^1^H NMR spectrum (CDCl_3_) of the ethylene oligomer from table 1, entry 1.

**Figure S24.** ^1^H NMR spectrum (CDCl_3_) of the ethylene oligomer from table 1, entry 2.

**Figure S25.** ^13^C NMR spectrum (CDCl_3_) of the ethylene oligomer from table 1, entry 3.

**Figure S26.** ^13^C NMR spectrum (CDCl_3_) of the ethylene oligomer from table 1, entry 6.

**Figure S27.** ^1^H NMR spectrum (CDCl_3_) of the ethylene oligomer from table 1, entry 9.

**Figure S28.** ^13^C NMR spectrum (CDCl_3_) of the ethylene oligomer from table 1, entry 9.

**Figure S29.** ^1^H NMR spectrum (CDCl_3_) of the ethylene oligomer from table 2, entry 1.

**Figure S30.** ^1^H NMR spectrum (CDCl_3_) of the ethylene oligomer from table 2, entry 2.

**Figure S31.** ^1^H NMR spectrum (CDCl_3_) of the E-MA co-oligomer from table 3, entry 1.

**Figure S32.** ^1^H NMR spectrum (CDCl_3_) of the E-MA co-oligomer from table 3, entry 2.

**Figure S33.** ^1^H NMR spectrum (CDCl_3_) of the E-MA co-oligomer from table 3, entry 3.

**Figure S34.** ^1^H NMR spectrum (CDCl_3_) of the E-MA co-oligomer from table 3, entry 4.

**3, References**

1. Yue, E.; Zhang, L.; Xing, Q.; Cao, X.-P.; Hao, X.; Redshaw, C.; Sun, W.-H., 2-(1-(2-Benzhydrylnaphthylimino)ethyl)pyridylnickel halides: synthesis, characterization, and ethylene polymerization behavior. *Dalton Transactions* **2014,** *43* (2), 423-431.

**4. X-ray Crystallography**


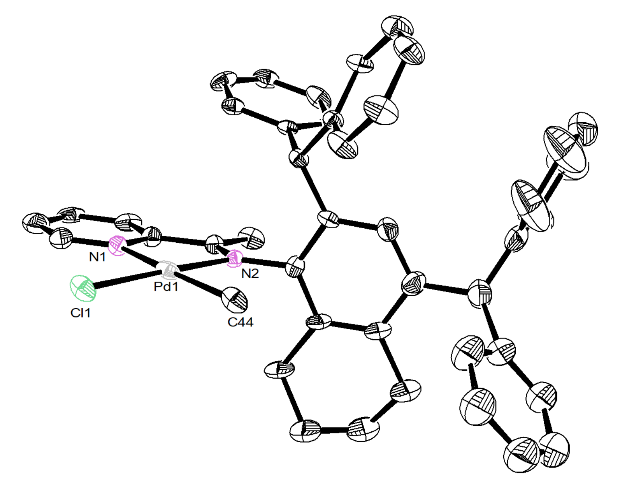


| **Table S1 Crystal data and structure refinement for Pd2.** | |
| --- | --- |
| Identification code | **Pd2** |
| Empirical formula | C44 H41 Cl N2 Pd |
| Formula weight | 739.69 |
| Temperature/K | 293(2) |
| Crystal system | Triclinic |
| Space group | P-1 |
| a/Å | 9.2957(9) |
| b/Å | 11.7588(11) |
| c/Å | 15.4097(14) |
| α/° | 87.540(3) |
| β/° | 87.042(3) |
| γ/° | 70.733(2) |
| Volume/Å^3^ | 1587.3(3) |
| Z | 2 |
| ρ_calc_g/cm^3^ | 1.405 |
| μ/mm^‑1^ | 0.699 |
| F(000) | 688 |
| Crystal size/mm^3^ | 0.40 x 0.20 x 0.08 |
| Radiation | MoKα (λ = 0.71073) |
| 2Θ range for data collection/° | 2.23 to 25.02 |
| Index ranges | -10<=h<=11, -8<=k<=13, -18<=l<=18 |
| Reflections collected | 7660 |
| Independent reflections | 5404 [R(int) = 0.0437] |
| Data/restraints/parameters | 5404 / 12 / 393 |
| Goodness-of-fit on F^2^ | 1.051 |
| Final R indexes [I>=2σ (I)] | R1 = 0.0646, wR2 = 0.1569 |
| Final R indexes [all data] | R1 = 0.0878, wR2 = 0.1671 |
| Largest diff. peak/hole / e Å^-3^ | 1.187 and -1.149 |
